# rMSIKeyIon: An Ion Filtering R Package for Untargeted Analysis of Metabolomic LDI-MS Images

**DOI:** 10.3390/metabo9080162

**Published:** 2019-08-02

**Authors:** Esteban del Castillo, Lluc Sementé, Sònia Torres, Pere Ràfols, Noelia Ramírez, Manuela Martins-Green, Manel Santafe, Xavier Correig

**Affiliations:** 1 Department of Electronic Engineering, Rovira i Virgili University, IISPV, 43007 Tarragona, Spain; 2 Spanish Biomedical Research Centre in Diabetes and Associated Metabolic Disorders (CIBERDEM), 28029 Madrid, Spain; 3 Department of Molecular, Cell and Systems Biology, University of California, Riverside, CA 92521, USA; 4 Unit of Histology and Neurobiology, Department of Basic Medical Sciences, Faculty of Medicine and Health Sciences, Rovira i Virgili University, Carrer St. Llorenç, No. 21, 43201 Reus, Spain

**Keywords:** mass spectrometry imaging, metabolomics imaging, biostatistics, ion selection algorithms

## Abstract

Many MALDI-MS imaging experiments make a case versus control studies of different tissue regions in order to highlight significant compounds affected by the variables of study. This is a challenge because the tissue samples to be compared come from different biological entities, and therefore they exhibit high variability. Moreover, the statistical tests available cannot properly compare ion concentrations in two regions of interest (ROIs) within or between images. The high correlation between the ion concentrations due to the existence of different morphological regions in the tissue means that the common statistical tests used in metabolomics experiments cannot be applied. Another difficulty with the reliability of statistical tests is the elevated number of undetected MS ions in a high percentage of pixels. In this study, we report a procedure for discovering the most important ions in the comparison of a pair of ROIs within or between tissue sections. These ROIs were identified by an unsupervised segmentation process, using the popular k-means algorithm. Our ion filtering algorithm aims to find the up or down-regulated ions between two ROIs by using a combination of three parameters: (a) the percentage of pixels in which a particular ion is not detected, (b) the Mann–Whitney U ion concentration test, and (c) the ion concentration fold-change. The undetected MS signals (null peaks) are discarded from the histogram before the calculation of (b) and (c) parameters. With this methodology, we found the important ions between the different segments of a mouse brain tissue sagittal section and determined some lipid compounds (mainly triacylglycerols and phosphatidylcholines) in the liver of mice exposed to thirdhand smoke.

## 1. Introduction

Mass Spectrometry Imaging (MSI) is a label-free analytical technique that can locate chemical compounds (metabolites, peptides, lipids, or proteins) directly in a biological sample and give their concentration for every pixel. The most common analytical strategy is MALDI due to its soft ionization, fast analysis, high throughput, versatility, and selectivity [1]. Other techniques, like desorption electrospray ionization (DESI), are becoming more popular because of the simplicity of their sample preparation [2]. MSI is currently used in the fields of drug discovery and toxicology [3,4]. In most experiments, researchers use a targeted strategy, which consists of visualizing and (sometimes) quantifying the concentration of a particular compound, or a reduced set of compounds throughout the tissue. Many MSI software packages have been released [5]. However, none of them provides an automated workflow for untargeted MSI applications since the end-user has to approach each MSI experiment data analysis in its unique manner.

Besides annotating and identifying the MS ions, one of the main challenges in untargeted MSI analysis is to determine the statistically differentiating ions in different regions of interest (ROIs) of the same tissue section or in different tissues of case versus control experiments. These key ions could be associated with biomarker candidates of disease or treatment efficacy. Previous studies have successfully used segmentation processes to find these key ions between clusters [6,7]. Most of these studies identify the key ions associated with a certain region by analysing the ions that most influence the segmenting process. In [8], the authors applied a Non-negative Matrix Factorization multivariate analysis to select a reduced group of lipid MS signals associated with the metabolite profile of each component. The *t*-test associated with segmentation with Spatial Shrunken Centroids can find the enriched and absent MS peaks for a particular region in a segmented image [9,10]. A technique based on deep unsupervised neural networks and parametric *t*-SNE was used to detect metabolic hidden sub-regions [11]. The same algorithm, linked to a significance analysis of microarrays (SAM), detected the protein subpopulations that can differentiate between *t*-SNE segments in a dataset of breast cancer samples; interestingly, they used the selected ions for a kNN second segmentation step [12]. Gorzolka et al. [13] studied the space-time profiling of the barley germination process by carrying out an unsupervised joint segmentation of a high number of images and found the ion-associated profile for every segment. The Algorithm for MSI Analysis by Semi-supervised Segmentation (AMASS) was used to segment leech embryo samples [14] and there is a complete analysis of the ions associated to every region according to its weighting factors. In all these references, no statistical significance test was conducted on the key ions found.

Another common strategy in MSI data analysis is to manually define the ROIs to be compared, guided by an annotated histology image [15,16,17,18]. In general, the ions are selected by means of statistical hypothesis testing and the fold change (FC) calculation of the ion concentrations between ROIs. These parameters are usually represented as volcano plots. By way of example, Hong et al. [19] studied the global changes of phospholipids in brain samples from a mouse model of Alzheimer disease by performing ANOVA tests of ion concentrations in ROI. A common problem that MSI has in calculating statistical significance is that the *p*-values are generally extremely low [16]. This is because there are a large number of pixels within each ROI, which gives this parameter a low discrimination power.

Additionally, the statistical hypothesis testing (such as the *t*-test) fails when is applied to compare the concentration of an ion between ROIs. The existence of morphological areas in the images is the responsible of a high pixel autocorrelation. This violates the assumption of observation independence necessary for statistical hypothesis testing. In order to find statistically significant ions between ROIs, Conditional Autoregressive (CAR) models, which take into account the auto-correlated nature of ion distribution concentration in MS image ROIs, are calculated to correct the *p*-values [20]. Nevertheless, the difficulty of calculating the autocorrelation models and the complexity of the computational approach hampers the inclusion of this strategy in a MSI workflow.

Another common situation in MS imaging is the elevated intensity differences of the ions’ concentration between pixels, due to the existence of several morphologic regions with different metabolic profiles [21] and the ion shielding phenomena which takes place in MSI. It is also common to find a high proportion of pixels where a certain ion is not detected, for a given signal to noise ratio. This influences to a large extend the calculation of the *p*-values and the FC.

In this study, we describe the development of an ion filtering algorithm that is used in a workflow for the untargeted analysis of metabolomic MALDI-MS images. The workflow consists of a segmentation step, followed by the ion filtering procedure, independent of the segmentation process, that detects the up/down regulated ions between image segments. Our algorithm calculates and combines three parameters: (a) the Mann–Whitney U statistical test of the ion concentration between segments [22]; (b) the FC in the ion concentration between segments; and (c) a new parameter that accounts for the proportion of pixels with undetected ions between segments. In addition, the data from which parameters (a) and (b) are derived is obtained by previously filtering out the undetected MS signals (null values). With this methodology, we can find the key ions between any segment pair in MSI datasets, from single or multiple tissue sections. We successfully applied this workflow to the analysis of mouse brain tissue sample and to study fatty liver disease in mice liver tissue samples.

## 2. Results

**Figure 1:**
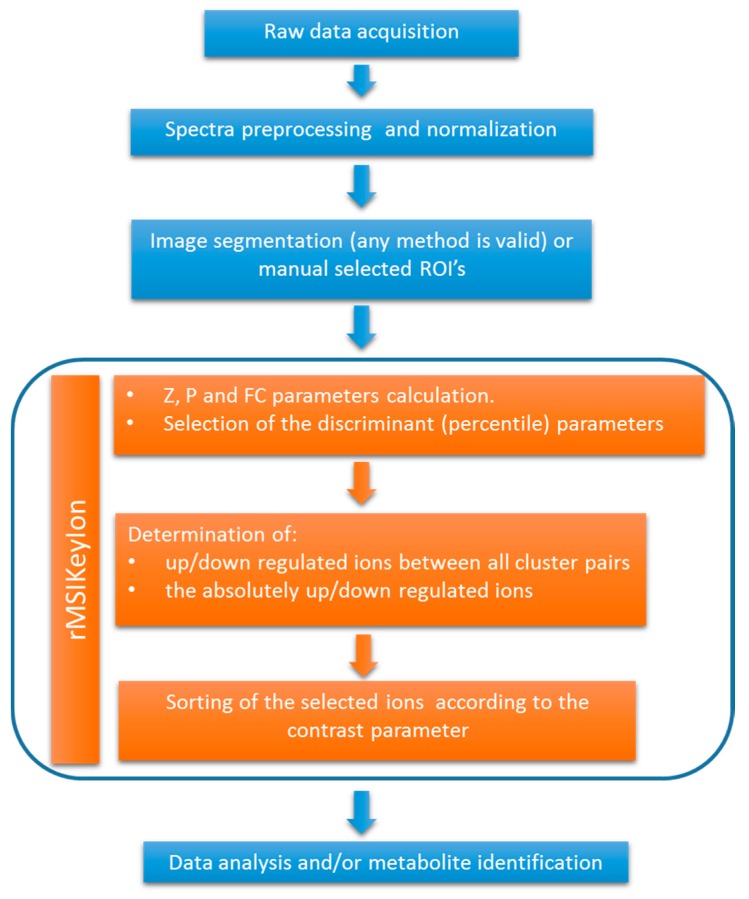
Workflow of the data processing, indicating the steps performed by the rMSIKeyIon package.

The rMSIKeyIon package, written in R, is able to find the key ions in a pair of ROIs within or between images. The ions are selected according to the similarity parameters calculated in Appendix A and ordered following the contrast parameter, described in Appendix B. In Figure 1, there is a description of the data processing workflow, showing the main steps implemented in the rMSIKeyIon package. The spectra preprocessing and image segmentation has to be performed before and independently to the rMSIKeyIon execution. The resulting list of selected ions is related to the key metabolites exhibiting biological difference between tissue regions and reducing the candidates to identify.

In the next section, we will describe the results of the package in the analysis of a sagittal brain mouse sample, which has been segmented by k-means algorithm (Section 2.1). In particular, we will illustrate the up or down regulated ions resulting of the comparison of two clusters and the up/down regulated ions when comparing one cluster with the rest.

In the Section 2.2, we will apply the package in the identification of the fat areas in control liver samples and liver samples exposed to thirdhand smoke (THS). 

### 2.1. Results of the Brain Mouse Sample

**Figure 2:**
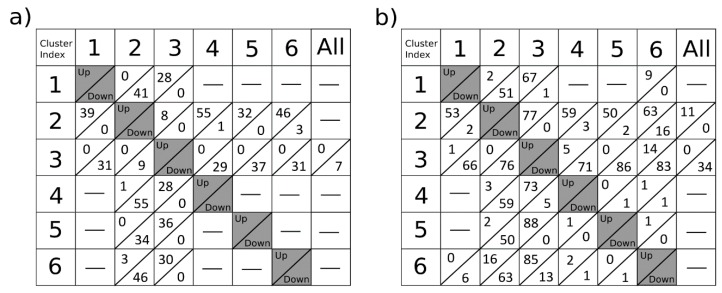
Number of up or down-regulated ions associated with the comparison of one particular cluster with each of the others (columns 1 to 6) and the ions that are up-regulated (or down-regulated) in a cluster as a result of the comparison between this cluster and the rest of clusters, called “absolutely up-regulated ions” (or “absolutely down-regulated ions”). The image is composed by 6898 pixels and the number of detected ions is 277. The percentile value used for the selection of the ions is 1% for the null concentration parameter (Z) and 10% for the Mann–Whitney U (V) test and for the concentration fold change (FC). The intensity threshold for the ions is 2.5 × 10^−4^ over the normalized spectra matrix. The small lack of symmetry observed in the table is a consequence of the lack of symmetry in the distributions considered. In (**a**), the up-down regulated ions are calculated following the classical procedure, while in (**b**) the ions are calculated according the procedure described in section methods, that considers that the null values are not taken into account.

Figure 2 shows the number of up and down-regulated ions associated with the comparison of one particular cluster with each of the others (columns 1 to 6) in the segmented image of the brain slice tissue of C57BL/6 mouse using the k-means algorithm (n = 7 clusters). Cluster 7, identified as non-tissue section areas, has not participated in the comparisons. In column “All” appear the ions that are up-regulated (or down-regulated) in a cluster as a result of the comparison between this cluster and the rest of clusters, called “absolutely up-regulated ions” (or “absolutely down-regulated ions”). The *m/z* values resulting from comparisons can be available at the GitHub repository of the package (https://github.com/LlucSF/rMSIKeyIon).

For each cluster comparison, an associated figure gives information about the resulting up or down-regulated ions, and the number of null and non-null parameters defined in the section Ion analysis and filtering (see below). The ions on the list are ordered in terms of the value of the “contrast parameter”, calculated with Equation (A4) in Appendix B.

Figure 2a shows the results obtained by the classical procedure, where null values do not have a special treatment. Figure 2b corresponds to the case in which the null values are treated separately. Although both cases make use of the same processing parameters, the results are very different. Figure 2b shows a higher abundance of up-down regulated ions versus Figure 2a. In addition, the ions find in Figure 2b are of higher relevance, as can be seen in Figure S1. Figure S1 shows the two ions with the highest contrast value from the volcano plot when comparing clusters 2 and 6. Figure S1a corresponds to the classic test, and Figure S1b corresponds to the separation of the null values.

A slightly asymmetry is displayed in the tables present in Figure 2. Each parameter has its own set of discriminant values. They are obtained from the evaluation of each parameter on all the pairs of clusters without repetition. The distribution generated by the set of all these values may not be symmetric. By applying the same percentile on both tails of the distribution, non-symmetric discriminant values may arise.

#### 2.1.1. Comparison of C2 & C6

By way of example, the comparison of clusters C2 and C6 showed 63 up-regulated ions in C2 versus C6 and 16 down-regulated ions in C2 versus C6.

As an example, Figure S2 shows the volcano plot of the ions resulting from the comparison of C2 and C6. The ions at the top right and top left are selected by the ion filtering algorithm (see the caption to Figure S2 for more details).

Figure S3a shows the histogram of the concentration of the up-regulated ion with the highest contrast parameter (*m/z* 198.076) in C6, and Figure S3b shows the histogram of the up-regulated ion (*m/z* 848.636) in C2 also with the highest contrast parameter.

**Figure 3:**
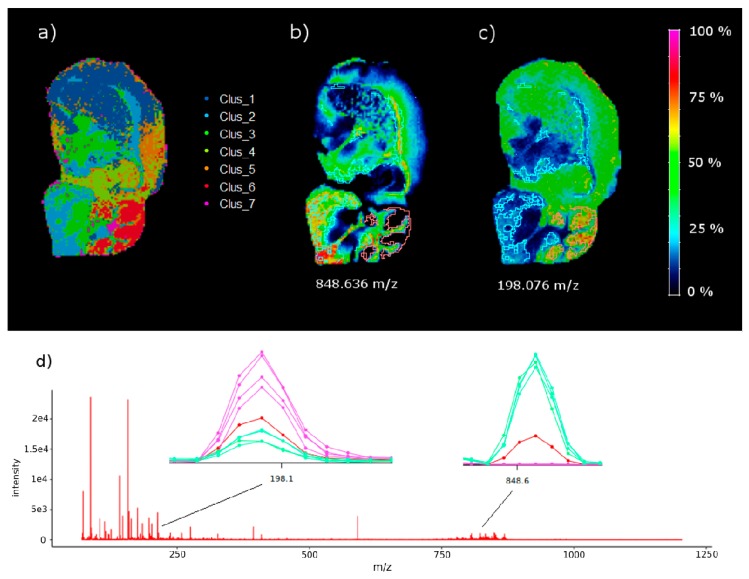
(**a**) Mouse brain segmentation using k-means (n = 7 clusters), (**b**) intensity map of ion *m/z* 848.636 (the up-regulated ion in C2 versus C6 with the highest contrasting parameter extracted from the null concentration parameter) and (**c**) intensity map of ion *m/z* 198.076, the down-regulated ion with the highest contrast parameter after comparing C2 and C6, extracted from the volcano plot. The highlighted areas in (**b**,**c**) represent C2 (white contour) and C6 (red contour). (**d**) Mean spectrum (red), spectra from C2 pixels (green), and spectra from C6 pixels (pink) near *m/z* 848.636 and *m/z* 198.076. The spectra show the up-regulated and down-regulated behaviour of the ions. See also the optical image of the same brain tissue section stained with a Hematoxilyn in Figure S4.

Figure 3a shows the segmented brain image (n = 7), and Figure 3b,c shows the concentration intensity plot of the ions mentioned above. In these intensity maps, the contrast intensity between both ions and clusters is clear, and the intensity of *m/z* 848.636 is much higher in C2 than in C6 and vice-versa for *m/z* 198.076.

#### 2.1.2. Absolutely Up and Down-Regulated Ions in Brain

**Figure 4:**
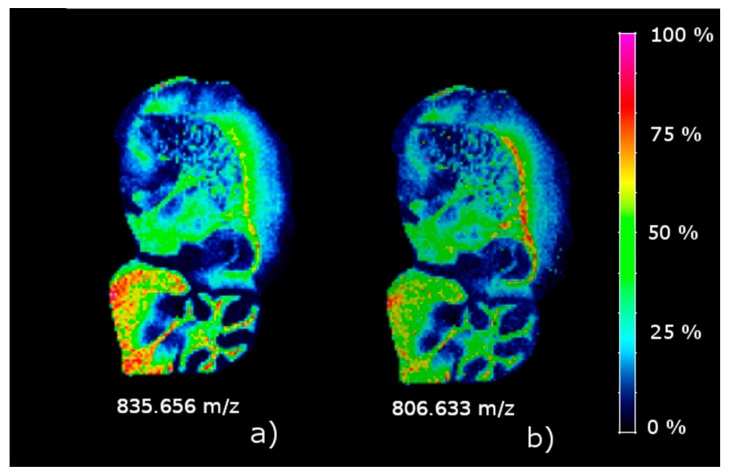
Concentration images of the two absolutely up-regulated ions in C2. (**a**) *m/z* 835.656; (**b**) *m/z* 806.633.

**Figure 5:**
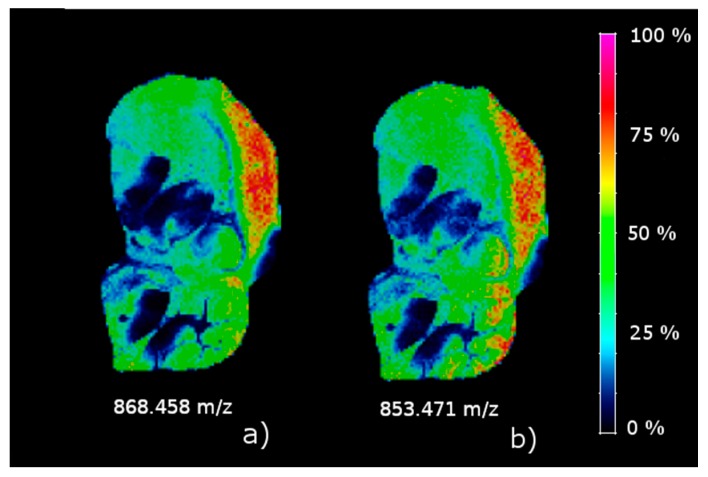
Concentration images of two absolutely down-regulated ions in C3. (**a**) *m/z* 868.458; (**b**) *m/z* 853.471.

According to the results in Figure 2b, there are 11 absolutely up-regulated ions in C2, and 34 absolutely down-regulated ions in C3. Figure 4 shows the concentration intensity plot of the two up-regulated ions (*m/z* 835.656 and *m/z* 806.633) in C2, and Figure 5 shows two down-regulated ions (*m/z* 868.459 and *m/z* 853.471) in C3 with the highest contrast parameter. There is an evident similarity between the images of the two up-regulated ions for one hand and two down-regulated ones for the other one. A comparison of the images in Figure 4 with the distribution of C2 in the brain are clearly similar. And the same is true of a comparison of the images in Figure 5 with the distribution of C3 in the brain.

### 2.2. Results of the Liver Samples

**Figure 6:**
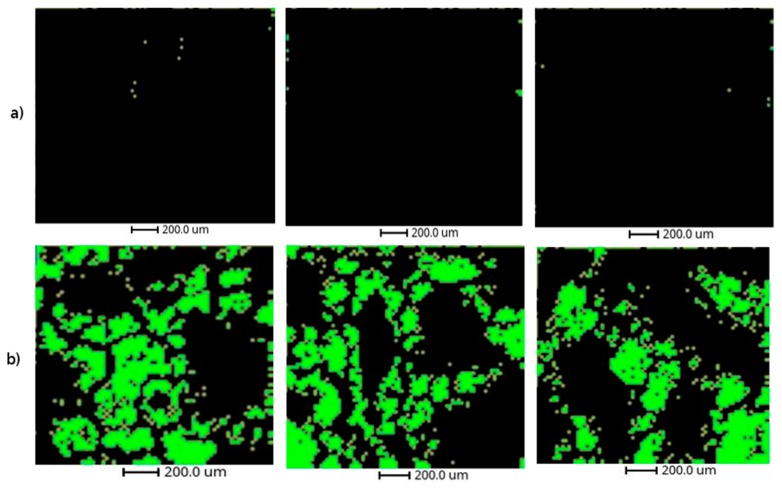
Representation of cluster 2 of the six liver samples: (**a**) the three analytical replicates of a control mouse and (**b**) the three replicates of a thirdhand smoke (THS)-exposed mouse.

The methodology used in this article has been applied to the study of non-alcoholic fatty liver disease in mice exposed to thirdhand tobacco smoke (TBS) [23]. We have taken a total of six images from the liver samples (three from a control mouse and three from a THS-exposed mouse). The images has been segmented using the k-means algorithm (n = 6 clusters). The results of rMSIKeyIon algorithm showed that cluster 2 (C2) has an elevated number of ions in the lipid mass range that are absolutely up-regulated, and we hypothesized that this cluster represents the lipid droplet areas characteristic of the fatty livers (see Figure 6) and the full segmented image (see Figure S5). The THS exposed mouse has the largest area, while the control animals have the smallest, in accordance with Martins-Green et al. [23]. In addition, the Figure S6 is an optical image of a selected area of a tissue section of a control and a THS exposed mouse stained with an Oil Red O protocol. It can also be observed the higher density of lipid droplets in the THS exposed sample.

Table S1 shows the compounds in C2 putatively identified after a manual curation process. As can be observed, most of them are putatively identified as triglycerides or phosphatidylcholine. In Figure S6, there is the intensity map of the triacylglycerol (50:30), which is highly similar to the geometry of C2.

## 3. Discussion

Here, we developed a new methodology for the untargeted analysis of MS images that can be used coupled with any segmentation process and an ion filtering algorithm based on the combination of three parameters: (a) The ratio of ions with a null concentration between the regions, (b) the U Mann–Whitney U Test, calculated by segregating the non-detected ions from the distribution, and (c) the FC between the medians of the distribution (the non-detected ions were also segregated from the distribution). This methodology has proved to be efficient at finding the up/down-expressed ions in an intra-image analysis or in the comparative analysis of groups of images. The presented workflow is different to previously released software tools due to two main reasons: (a) it is flexible and independent to the segmentation process, so the ion selection process can be applied to any clustering algorithm or manually drawn ROIs. (b) Our methodology provides a completely automated ion filtering approach enabling the fast detection of a morphological region characteristic ions.

The results on the sagittal mouse brain sample show that an unsupervised clustering process followed by the rMSIKeyIon algorithm is able to select the (possible) up/down-regulated ions between any pair of clusters, in a holistic approach, and between one cluster and the rest. The concentration maps of the selected ions, ordered by the contrast parameter, depicts faithfully the morphology of the brain. These ions are probably biologically relevant and could be interesting to identify.

Using the described methodology, we have been able to detect the regions containing the lipid droplets in the liver samples from mouse exposed to THS. The putative identification of the key up-regulated ions in the cluster 2, mainly triglycerides and phosphatidylcholines, confirm that THS exposure conducts to the apparition of fatty liver disease in mice [23].

Untargeted metabolomics data analysis workflows are associated to standard analytical platforms (LC-MS, GC-MS, and NMR) [24]. These analyses compare the concentrations of chemical compounds in a CASE and a CONTROL group in order to discover features that they express differently and which could be used as biomarkers or in biological pathway analysis. In general, the number of samples (n) of each experimental group are similar, the distribution is normal (for large n values), and the principle of independent measures is assumed. However, in spatial metabolomics, the number of samples in every group (i.e., the number of pixels in an ROI) is not determined a priori, as in metabolomics studies.

Untargeted image analysis has two main applications:

(a) The comparison of two regions inside the same tissue section (intra-image analysis) to find the relevant ions. This could be used to discover cancer biomarkers by comparing the ion profile of the tumorous area with a non-tumorous area from the same sample. In general, the areas to be compared are determined by a histopathologist annotating a consecutive tissue section. The size of the ROIs in which we will compare the ions is determined manually.

(b) For several reasons, the analysis of morphologically equivalent regions in different tissues in a case-control experiment is much more complicated. First of all, the tissue samples to be compared between groups are equivalent but not similar because of the biological differences between the animals and the intrinsic difficulty of achieving identical tissue sections. Consequently, it is not straightforward to delimit the areas to be compared. The ROIs to be compared can be determined by histological annotation (supervised process), or automatically by means of a segmentation process (unsupervised process). In both cases, there are not established rules, and the following steps in the statistical analysis of the ions between ROIs can be highly affected by this fact.

In both cases, it is very common to find skewed ion distributions and a high percentage of null values, a high degree of autocorrelation between pixels, and a very high number of observations (pixels). This leads to extremely low *p*-values when classical parametric or non-parametric statistical tests are used [25], so these tests are not appropriate for this kind of analysis. For all the above reasons, the untargeted analysis of images remains a challenge. However, the results shown by rMSIKeyIon R package have been revealed to be very useful to find the most differential ions between ROIs. The biological relevance of these ions has been validated in a fatty liver study with animal models.

## 4. Materials and Methods

### 4.1. Materials

Indium tin oxide (ITO)-coated glass slides were obtained from Bruker Daltonics (Bremen, Germany). The gold target used for sputtering coating was obtained from Kurt J. Lesker Company (Hastings, England) with a purity grade higher than 99.995%. HPLC grade xylene was supplied by Sigma–Aldrich (Steinheim, Germany), and ethanol (96% purity) was supplied by Scharlau (Sentmenat, Spain).

### 4.2. Methods

#### 4.2.1. Sample Preparation

Mice models were developed at the Department of Molecular, Cell, and Systems Biology at the University of California Riverside [23]. Animal experimental protocols were approved by the University of California, Riverside, Institutional Animal Care and Use Committee (IACUC). The animal use protocol is A3400-01. The suitability of the workflow presented here to determine significant ions between ROIs from the same tissue was tested in a brain sample from a 6-month-old C57BL/6 mouse feed with a standard chow diet (percent calories: 58% carbohydrates, 28.5% protein, and 13.5% fat). To test the suitability of the method in different tissue sections in a case versus control experiment, we used liver samples from mice exposed to THS—the residual particles and gases from tobacco smoke that remain in dust and surfaces—from weaning (three weeks of age) to 24 weeks, without exposure to secondhand smoke (SHS) at any time during the study, and compared them with liver samples of mice that had not been exposed to THS (control group) [26]. Brain and liver samples were snap frozen at −80 °C after collection and stored and shipped at this temperature until analysis.

For MSI acquisition, the tissues were sectioned at −20 °C in slices 10 µm thick using a Leica CM-1950 cryostat (Leica Biosystems, Nussloch, Germany) located at the Centre for Omics Sciences (COS) of the Rovira i Virgili University and mounted on ITO slides by directly placing the glass slide onto the section at ambient temperature. To remove residual humidity, samples were dried in a desiccator under vacuum for 15 min after tissue mounting.

#### 4.2.2. Deposition of Au Nanolayers for LDI-MS Imaging

Gold nanolayers were deposited on the 10 µm tissue sections using an ATC Orion 8-HV sputtering system (AJA International, N. Scituate, MA, USA) [27]. Briefly, an argon atmosphere with a pressure of 30 mTor was used to create the plasma in the gun. The working distance of the plate was set to 35 mm. Sputtering conditions for MS were ambient temperature, and RF mode at 60 W for 50 s. The argon ion current was adjusted to 20 mL min ^−1^.

#### 4.2.3. LDI-MS Acquisition

One image of a sagittal brain tissue section and six liver tissue sections (three slices from a control animal and three sections from a THS-exposed animal) were acquired using a MALDI TOF/TOF UltrafleXtreme instrument with SmartBeam II Nd:YAG/355 nm laser from Bruker Daltonics, also at the COS facilities. Raster sizes of 80 and 20 µm were used for the brain and liver tissue sections, respectively. The TOF spectrometer operated in reflectron positive mode with the digitizer set at a sample rate of 1.25 GHz in a mass range between 70 and 1200 Da. The spectrometer was calibrated prior to tissue image acquisitions using [Au]^+^ cluster MS peaks as internal mass references [27].

#### 4.2.4. MSI Data Processing and Image Segmentation

The MSI data acquired with Bruker’s FlexImaging 3.0 software was exported to XMASS data format using instrument manufacturer software packages (FlexImaging and Compass export). The raw data was loaded using the in-house rMSI package [28]. This package provides a data storage format based on segmented matrices and optimized for processing large MSI datasets in R language. Next, we applied our complete MSI pre-processing workflow consisting of spectral smoothing, alignment, mass recalibration, peak detection and peak binning [29] with the default parameters: Savitzky–Golay kernel size of 7, peak detection threshold SNR of 5, and peak binning tolerance of 6 scans with 5% filter. At this point, we obtained a peak matrix object of each MSI dataset: the brain tissue sagittal section and the liver tissue sections. These peak matrix objects are highly reduced, robust, and accurate representations of all the MSI data and can be used to perform complex statistical analyses on the huge amount of data generated in the MSI experiment. ROIs were generated by means of a k-means process. Finally, we applied the rMSIKeyIon workflow using the peak matrices as the input data.

#### 4.2.5. Ion Analysis and Filtering

The procedure used for identifying statistically different ions compared the concentration distributions of the ions in all possible pairs of ROIs in which the tissue (or tissues) had been segmented.

In general, the total number of pixels in each ROI is different and the probability density function of the ion concentrations is not normal. We used the Mann–Whitney U test [22] because it can test the null hypothesis (both sets of samples come from the same distribution) of two non-normal distributions that have a different number of observations.

In addition, in non-normal distributions of different sample sizes, there is usually a singular element: In some ROIs, there is a considerable possibility that the distribution of some ions will have small concentration values. Figure S8 represents the percentage of non-detected ions in the segmented brain image, using the k-means algorithm with n = 7 clusters. It can be observed that for some clusters (i.e., cluster 7) the percentage is very high.

For purposes of illustration, Figure S9 shows a simulated histograms of an ion in two different clusters with samples taken from normal distributions, with different average values, to which significant amounts of null values have been added. In total, there are 200 samples for both cases. Both distributions appear to be very different and the Mann–Whitney U test yields a very high *p*-value (0.38). The idea we have worked on here is to segregate the values obtained from non-detected ions (null values) from the rest of the distribution so that they can be treated separately. Thus, we obtain a very small *p*-value (of the order of 1 × 10^−43^). On the other hand, the percent of null values in each ROI also provides valuable information. For these reasons, we decided to segregate the null values from the ion matrix and use them to calculate a parameter (null concentration parameter), as will be explained below.

The calculation of the null concentration parameter, as well as the non-null parameters (Mann–Whitney U distribution and FC), are described in Appendix A.

Once the ions were selected using the two procedures described above, they were ordered in terms of the contrast generated by every ion between one ROI and the set of other ROIs. The procedure is described in Appendix B.

The ion filtering algorithm described in this section has been implemented in the R package named rMSIKeyIons, accessible at (https://github.com/LlucSF/rMSIKeyIon). The software’s source code was written in C++ and requires the GNU Scientific Library (GSL) (https://www.gnu.org/software/gsl). Later, it was ported to R using the Rcpp R package. As input, the function requires an rMSIproc peak matrix, a previously calculated segmentation and the percentiles for each parameter, and as output, the function returns a list containing the ions for each comparison between all pair of clusters and the data related with those ions.

#### 4.2.6. Metabolite Identification

The obtained list of up regulated lipids for mice liver samples in cluster 2 was matched with the HMDB 4.0 [30] database within a tolerance of 20 ppm and the possible ion adducts: H, Na, K, and NH4. Results were filtered using the biological information of molecules provided by the HMDB, thus metabolites with no biological origin or not likely to be found in liver were discarded.

## 5. Conclusions

In this study, we developed the ion filtering R package rMSIKeyIon. It is open source, publicly available, and based on the combination of three parameters: the non-detected ion concentration ratio, the Mann–Whitney U ion concentration test, and the FC in the ion concentration. The null values were discarded before computing the last two parameters.

We demonstrated that our tool is very effective at discovering up or down-regulated ions between clusters using an unsupervised k-means procedure. The ions selected are the candidates that, subsequently, have to be identified. This package is a valuable tool for the untargeted analysis of MALDI images and is an important advance in this area because, at present, there are no tools available.

## Supplementary Materials

The following are available online at https://www.mdpi.com/2218-1989/9/8/162/s1. The brain dataset, the used clustering and a R script containing instructions about the installation and the testing of the package accompanied with a document containing illustrative figures. Also the results of the method are included.

## Appendix A. Calculation of the Similarity Parameters between ROIs

In order to determine the ions that are expressed differently in two given ROIs, we calculate three parameters:

(a) The null concentration parameter (Z parameter)

The $Z_{ijk}$ parameter is calculated according to Equation (A1):

(A1) $$Z_{ijk}=\frac{\frac{Nz_{ij}}{N_{j}}}{\frac{Nz_{ik}}{N_{k}}}\forall i\in I;\forall j,k\in S_{p},$$

where $Z_{ijk}$ is the parameter that accounts for the null values (i.e., the non-detected values) of the *i* ion when comparing the *j* and *k* ROIs; $Nz_{ij}$ and $Nz_{ik}$ are the number of pixels with null values of the *i* ion in *j* and *k* ROIs, respectively; $N_{j}$ and $N_{k}$ are the total number of ROI pixels in *j* and *k*, respectively; *I* is the set of ions and *Sp* is the set of ROIs.

The equation calculates the ratio between the null values of a particular ion in the two ROIs. A value of $Z_{ijk}>Z_{high}$ ($Z_{high}$ being a positive value greater than 1) means that the *i* ion is more expressed in *k* ROI than in *j* ROI, while $Z_{ijk}<Z_{low}$ ($Z_{low}$ being a positive value much lower than 1) means that the *i* ion is less expressed in *k* ROI than in *j* ROI.

The importance of this parameter is assessed in Figure S7. For clusters 1 to 7, we plotted, the percentage of pixels that have null concentration for every ion.

The $Z_{high}$ and $Z_{low}$ values are calculated by following these steps:

(1) The *Z* values of all ions, for all cluster-pairs, are calculated according to Equation (A1).
(2) An ordered rank list of all the *Z* values is created.
(3) $Z_{low}$ is determined considering that this value is a certain percentile *P_Z_* of the rank list of Z values.
(4) $Z_{high}$ is determined considering that this value is a certain percentile 100 − *P_Z_* of the rank list of Z values.

(b) Non-null concentration parameters (V parameters)

Provided that the distribution of the ions concentration is non-normal, we considered the *U* Mann–Whitney U test and the concentration FC between two ROIs, as a non-null concentration parameters.

Generally speaking, if *N_j_* and *N_k_* are high, the random variable *U* can be regarded as normally distributed [22]. The $U_{ijk}$ parameter is then normalized following Equation (A2):

(A2) $$V_{ijk}=\frac{U_{ijk}-m_{u}}{\sigma_{u}},$$

where $m_{u}$ and $\sigma_{u}$ are the average and standard deviation of zero $U_{ijk}$ and $V_{ijk}$ is a random variable with a normalized Gaussian distribution. If *V* has values close to 1 the similarity between the distributions is high, while values close to zero indicate disparate distributions. The value obtained for *V* indicates the similarity between the distributions of two ROIs for an ion.

Another parameter often used to compare sets of magnitudes is the FC, defined as the ion median concentration quotient between two ROIs Equation (A3):

(A3) $$FC_{ijk}=\frac{M_{ij}}{M_{ik}},$$

where $M_{ij}$ is the distribution median of the *i* ion in *j* ROI and $M_{ik}$ is the same for *k* ROI. For every *i* ion, the $FC_{ijk}$ parameter is calculated between the *j* and *k* ROIs. For a pair of ROIs, a Volcano plot [31] can be drawn from the *V* and *FC* parameters.

In this representation, the position occupied by the ions is important: the ions located in the top corners generate very different distributions in the two ROIs. The ions at the top left are under-expressed ($V_{ijk}<V_{high}\wedge Fc_{ijk}<Fc_{low}$) and the ions at the top right are over-expressed ($V_{ijk}<V_{high}\wedge Fc_{ijk}>Fc_{high}$).

The values $V_{high}$, $Fc_{high}$ and $Fc_{low}$ are calculated following the same steps as for $Z_{high}$ and $Z_{low}$, but with a difference in the percentile value. The ions located in the areas of interest must satisfy the probability of being within a range associated with two random variables; that is to say:

$P\left(V_{ijk}\leq V_{high},Fc_{ijk}\leq Fc_{low}\right)$ for under-expressed ions and $P\left(V_{ijk}\leq V_{high},Fc_{ijk}\geq Fc_{high}\right)$ for over-expressed ions. Assuming that these are independent random variables, we obtain $P\left(V_{ijk}\leq V_{high}\right)=P\left(Fc_{ijk}\leq Fc_{low}\right)=P\left(Fc_{ijk}\geq Fc_{high}\right)=\sqrt{P_{z}/100}$. That is, the percentile that has to be used to determine the cutoff values in the volcano plot should be $P_{V}=10\cdot \sqrt{P_{Z}}$

## Appendix B. Determination of the Discriminating Figure Values and Generation of the Discriminant Ions Lists

The contrast parameter $C_{ij\vee S_{p}}$ of the *i* ion between the *j* ROI and all the ROIs ($\mathrm{set}\;S_{p}$ is calculated according to Equation (A4)):

(A4) $$C_{ij\vee S_{p}}=\frac{\frac{1}{N_{j}}\sum_{p=1}^{N_{j}}m_{ip}^{j}}{\frac{1}{N}\sum_{k=0}^{N_{S_{p}}}\sum_{p=1}^{N_{k}}m_{ip}^{k}},$$

where *N* is the total number of pixels in $S_{p}$, $N_{j}$ and $N_{k}$ are the number of pixels in the *j* and *k* ROIs respectively. $N_{S_{p}}$ is the total number of ROIs in set $S_{p}$, $m_{ip}^{j}$ and $m_{ip}^{k}$ are the magnitude of the *i* ion in pixel *p* of the *j* and *k* ROI, respectively. The list is ordered according to the $C_{ij\vee S_{p}}$, assuming that high values mean high contrast and vice-versa.
