# Supplementary material for: rMSIKeyIon: An Ion Filtering R Package for Untargeted Analysis of Metabolomic LDI-MS Images
Source: Metabolites. 2019 Aug 2;9(8):162. doi: 10.3390/metabo9080162 (PMC6724114; doi:10.3390/metabo9080162)
Supplement: Supplementary file 1 [file metabolites-09-00162-s001.pdf]

rMSIKeyIon: an ion filtering R package for untargeted analysis of metabolomic LDI-MS images

Supplementary information

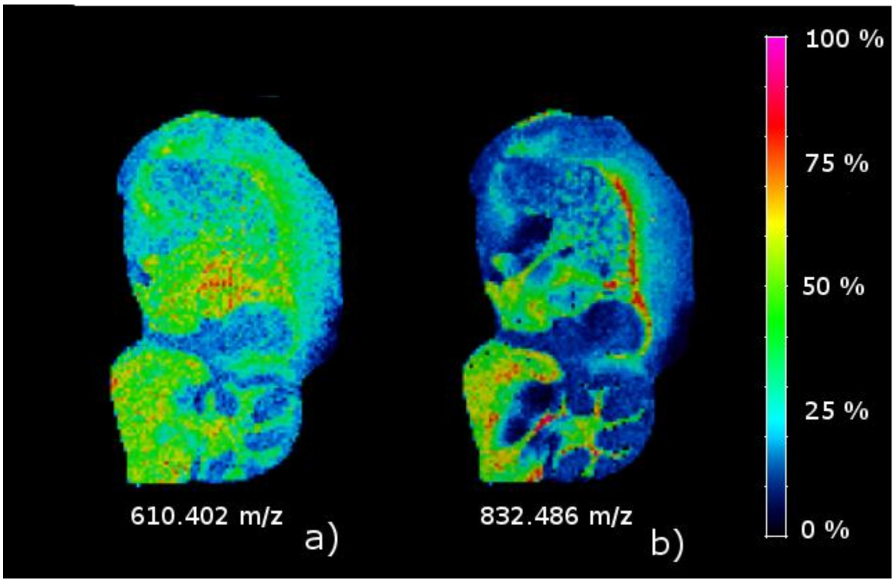

Figure S1, showing the concentration of the m/z 610.402 in (a) and m/z 832.486 in (b). The first (a), is the ion having a highest contrast value when comparing C2 vs C6 using the classical procedure (volcano plot). The second (b) is the ion, selected by volcano plot, with highest contrast value comparing C2 vs C6, using the procedure described in this work.

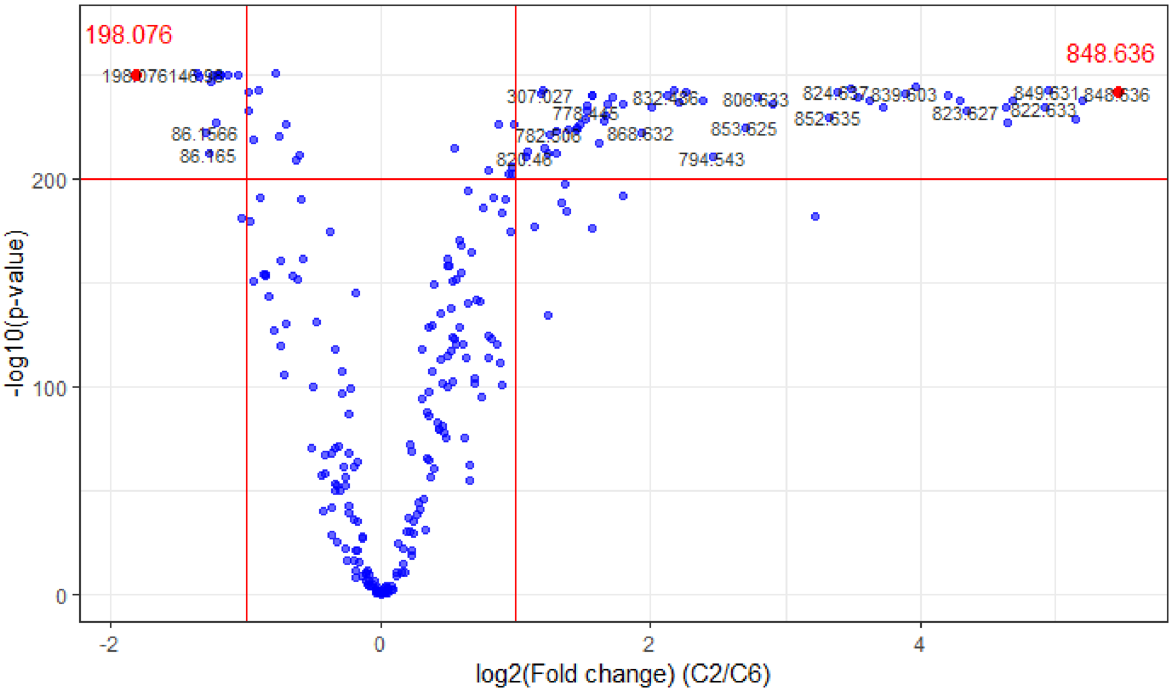

Figure S2. Volcano plot of the comparison between C2&C6. Ions in the right zone are up-regulated in C2 and the ones in the left zone are up-regulated in C6. The ions that have a lower p-value are placed in the upper part of the figure. We highlighted in red m/z 848.636, the ion up-regulated in C2 having the highest contrast parameter and the ion m/z 198.076, the ion up-regulated in C6 having also the highest contrast parameter.

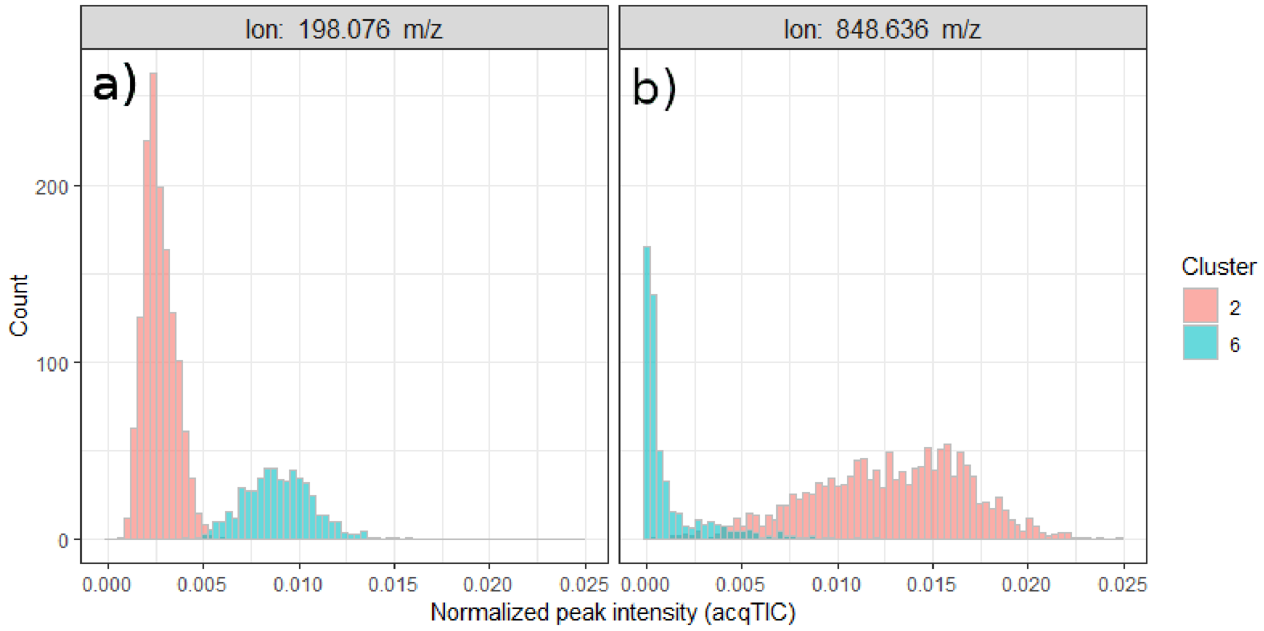

Figure S3. Histograms of ions selected in the volcano plot of the comparison between C2&C6 (see Fig. S1): m/z 198.076 (up-regulated in C6) and m/z 848.636 (up-regulated in C2). As can be appreciated, both distributions are dissimilar.

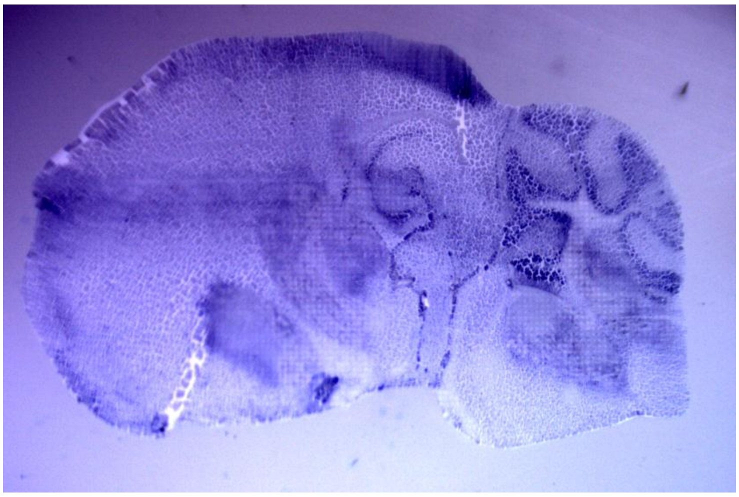

Figure S4. Histological images of the brain.

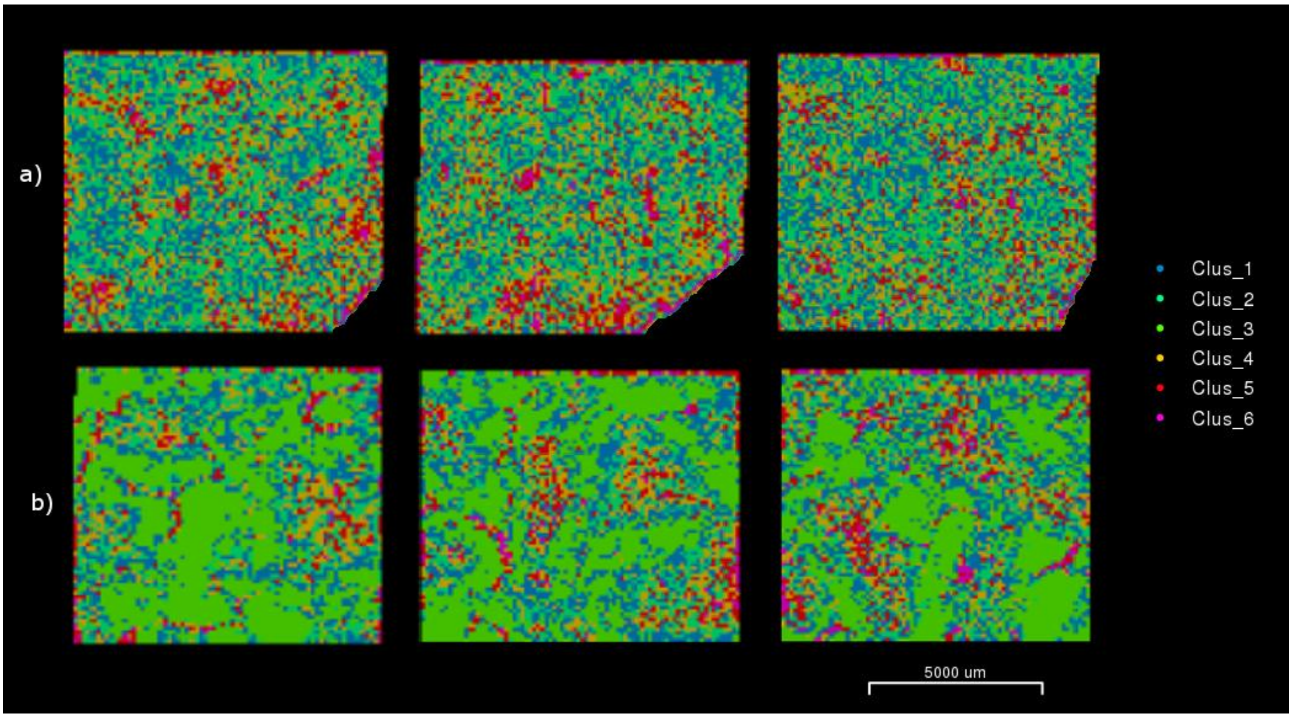

Figure S5. Liver segmented image with n=6 clusters

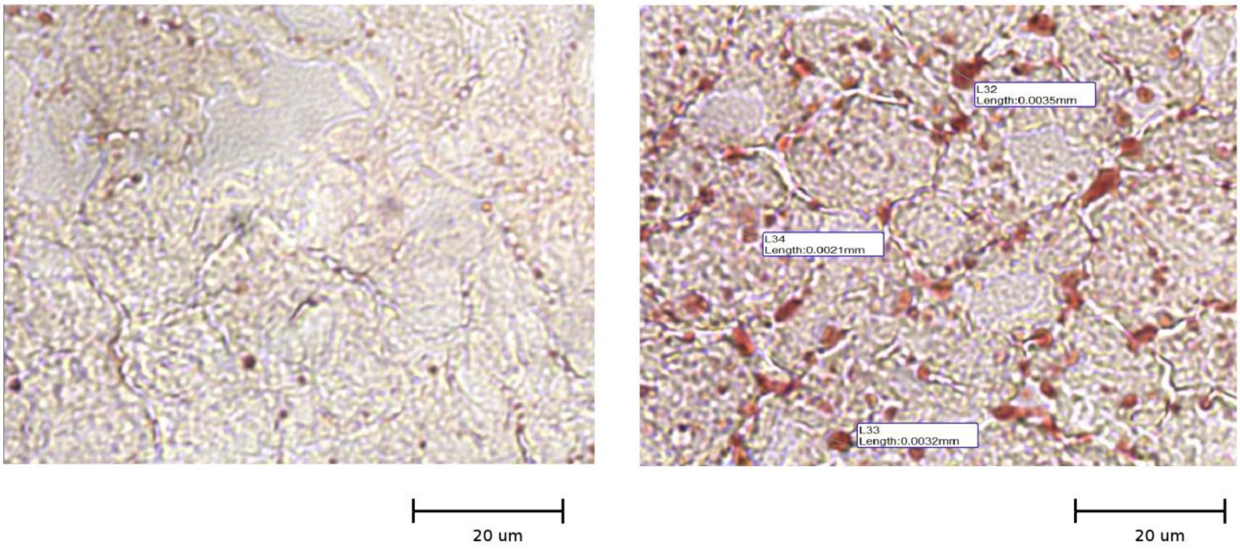

Figure S6. Histological images of liver with lipid droplets.

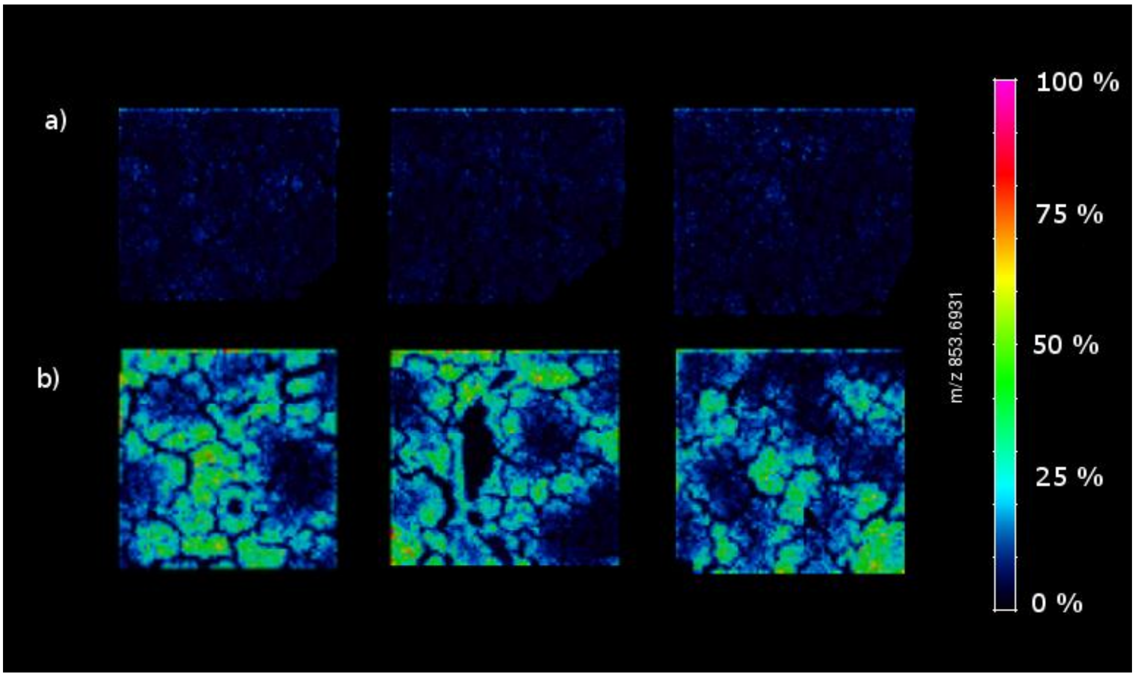

Figure S7. Intensity map of Triacylglycerol (50:30) concentration in the six liver samples: a) The three analytical replicates of a control mouse and b) The three replicates of a THS exposed mouse

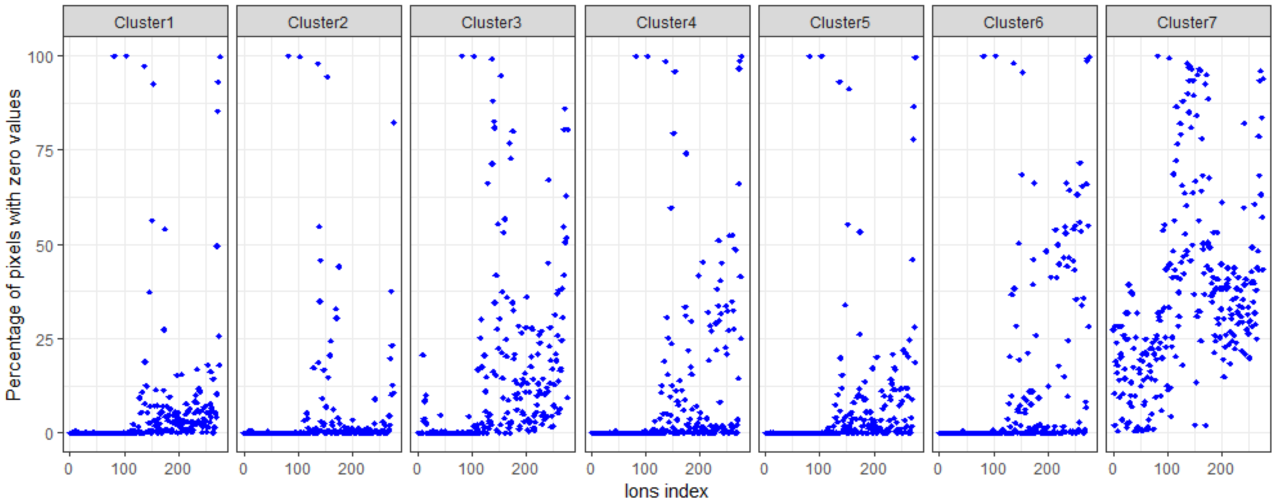

Figure S8. Images showing the ion's % of non-detected ions for segments 1 to 7 in the brain tissue section segmented with the k-means algorithm.

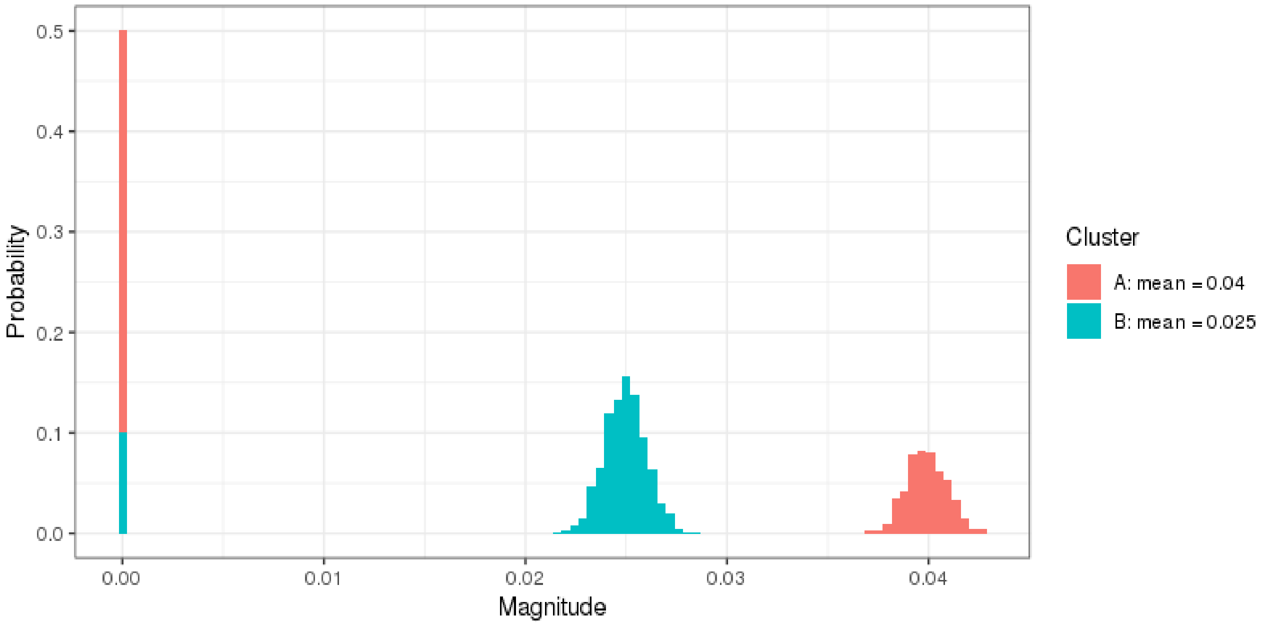

Figure S9. Synthetic histograms of one variable (i.e. m/z feature) in two clusters calculated with 200 observations (pixels) for each. The values were obtained from random sampling on normal distributions of means 0.025 and 0.04. The data of a sample includes 10% of null values; the data of the other sample includes 50% of null values (non-detected ions). Both histograms appear very different however the p-value indicates the opposite ($p = 0.38$) However, if we segregate the null values (non-detected ions), then the p value = $1e^{-43}$.

Name	Ion Formula	m/z exp	m/z calc	⚜m/z (ppm)
**Triacylglycerol(50:3)**	$[C_{53}H_{98}O_5+K]^+$	853.699	853.705	6.5
Phosphatidylcholine(44:3)	$[C_{52}H_{100}NO_7P+H]^+$	882.732	882.731	-1.1
Phosphatidylcholine(24:2)	$[C_{50}H_{96}NO_7P+H]^+$	854.704	854.700	-5.0
**Tracylglycerol(52:4)**	$[C_{55}H_{100}O_5+K]^+$	879.712	879.720	9.4
**Tracylglycerol(56:11)**	$[C_{59}H_{92}O_6+H]^+$	897.697	897.697	-0.4
Phosphatidylcholine(44:4)	$[C_{52}H_{98}NO_7P+H]^+$	880.716	880.715	-0.7
Tracylglycerol(50:4)	$[C_{53}H_{96}O_5+K]^+$	851.686	851.689	3.4
Tracylglycerol(52:2)	$[C_{55}H_{104}O_5+K]^+$	883.736	883.752	17.6
Phosphatidylcholine(42:0)	$[C_{50}H_{102}NO_7P+K]^+$	898.701	898.703	1.7
**Triacylglycerol(54:11)**	$[C_{57}H_{88}O_6+H]^+$	869.669	869.665	-4.2
**Phophatidylethanolamine(46:5)**	$[C_{51}H_{92}NO_8P+NH_4]^+$	895.686	895.690	4.3
Phosphatidylcholine(24:1)	$[C_{50}H_{98}NO_7P+H]^+$	856.716	856.715	-0.7
Phosphatidylcholine(42:0)	$[C_{50}H_{100}NO_7P+K]^+$	896.69	896.687	-3.5
Triacylglycerol(54:4)	$[C_{57}H_{104}O_5+K]^+$	907.745	907.752	7.2
**Triacylglycerol(52:7)**	$[C_{55}H_{92}O_6+Na]^+$	871.679	871.679	-0.4
Phosphatidylcholine(40:0)	$[C_{48}H_{96}NO_7P+Na]^+$	852.689	852.682	-8.6
Phosphatidylcholine(40:0)	$[C_{48}H_{98}NO_7P+K]^+$	870.672	870.671	-0.9
Triacylglycerol(52:5)	$[C_{55}H_{98}O_5+K]^+$	877.699	877.705	6.4
**Triacylglycerol(54:7)**	$[C_{57}H_{96}O_6+Na]^+$	899.703	899.710	7.7
**Tracylglycerol (55:7)**	$[C_{58}H_{98}O_6+NH_4]^+$	908.752	908.770	20.0
**Ceramide (t18:0/16:0)**	$[C_{34}H_{69}NO_4+Na]^+$	578.512	578.512	-0.2
Triacylglycerol(48:2)	$[C_{51}H_{96}O_5+K]^+$	827.685	827.689	4.8
SM (D18:0/23:0)	$[C_{46}H_{95}N_2O_6P+Na]^+$	825.67	825.682	14.5
Phosphatidic acid(46:0)	$[C_{49}H_{97}O_8P+Na]^+$	867.679	867.681	2.7
**Triacylglycerol(52:4)**	$[C_{55}H_{98}O_6+K]^+$	893.685	893.699	16.2
Triacylglycerol(54:5)	$[C_{57}H_{102}O_5+K]^+$	905.73	905.736	6.5
**4-hydroxy-3-(sulfooxy)/2-hydroxy-3-(sulfooxy) benzoic acid**	$[C_7H_6O_7S+H]^+$	234.987	234.9907	15.7

Table S1. Putative identification, by exact mass, of the absolutely up-regulated lipids in Cluster 2 (lipid droplets) of the 9 liver samples. In the table there is the name and the chemical formula of the lipids, as well as the mass error considered.
